# Using Lymphocyte and Plasma Hsp70 as Biomarkers for Assessing Coke Oven Exposure among Steel Workers

**DOI:** 10.1289/ehp.10104

**Published:** 2007-08-24

**Authors:** Xiaobo Yang, Jinping Zheng, Yun Bai, Fengjie Tian, Jing Yuan, Jianya Sun, Huashan Liang, Liang Guo, Hao Tan, Weihong Chen, Robert M. Tanguay, Tangchun Wu

**Affiliations:** 1 Department of Environmental and Occupational Health and Ministry of Education Key Lab for Environment and Health, School of Public Health, Tongji Medical College, Huazhong University of Science and Technology, Wuhan, China; 2 Department of Toxicology, Shanxi Medical University, Taiyuan, China; 3 Center for Disease Control and Prevention, Taiyuan Steel and Iron Limited Co., Taiyuan, China; 4 Laboratory of Cellular and Developmental Genetics, Department of Medicine, Faculty of Medicine, and CREFSIP (Centre de Recherche sur la Fonction, la Structure et l'Ingénierie des Protéines), Université Laval, Québec, Canada

**Keywords:** biomarker, coke oven workers, 1-hydroxypyrene, lymphocyte Hsp70, plasma Hsp70

## Abstract

**Background:**

Hsp70, an early-response protein induced when organisms are confronted with simple or complicated environmental stresses, can act as either a cellular protector or a danger signal.

**Objectives:**

The goal of this study was to evaluate levels of lymphocyte and/or plasma Hsp70 as biomarkers for assessing exposure response to complex coke oven emissions (COEs).

**Methods:**

We recruited 101 coke oven workers and determined levels of polycyclic aromatic hydrocarbon (PAH) exposure, urinary 1-hydroxypyrene (1-OHP), genotoxic damage by comet assay and micronuclei test, and other markers of damage, including plasma malondialdehyde (MDA) and lactate dehydrogenase (LDH). These were compared to levels of lymphocyte (intra-cellular) and plasma (extracellular) Hsp70 using Western blots and enzyme-linked immunosorbent assays (ELISA), respectively.

**Results:**

We observed a COEs-related dose-dependent increase in levels of DNA damage, micronuclei rate, MDA concentration, and LDH activity. Lymphocyte Hsp70 levels increased in the intermediate-exposure group (1.39 ± 0.88) but decreased in the high-exposure group (1.10 ± 0.55), compared with the low-exposure group. In contrast, plasma Hsp70 levels progressively increased as the dose of exposure increased. Negative correlations were seen between lymphocyte Hsp70 levels and olive tail moment and LDH activity in the intermediate- and high-exposure groups. However, we observed positive correlations between plasma Hsp70 levels and LDH activity in the low and intermediate groups.

**Conclusions:**

In workers exposed to COEs, high lymphocyte Hsp70 levels may provide protection and high plasma Hsp70 levels may serve as a danger marker. Larger validation studies are needed to establish the utility of Hsp70 as a response marker.

Coke oven workers are exposed to various occupational stressors, such as high carbon monoxide, high temperature, and toxic chemical substances including polycyclic aromatic hydrocarbons (PAHs) that are released into the workplace when coal is pyrolyzed. Exposure to PAHs has been shown to result in a dose-dependent risk of cancer at various organ sites, such as lung, skin, and bladder in humans (Mastrangelo et al. 1996). It has been well established that genomic instability phenotypes, possibly resulting from unrepaired DNA damage, lead to the initiation and progression of cancer (Fenech 2002; Smith et al. 2003). In addition, metabolic transformation of PAHs generates reactive electrophilic metabolites, causing DNA damage (Wang et al. 2002) and triggering the production of reactive oxygen species that lead to oxidative stress and the induction of many proteins, including heat shock proteins (Hsps) (Burczynski et al. 1999; Lee and Corry 1998).

Hsps are molecular chaperones whose role is to maintain normal cellular functions by binding to unfolded or misfolded proteins, promoting either refolding or proteolytic degradation of these proteins, thereby protecting cells against protein aggregation (Jindal 1996; Nollen et al. 1999; Young et al. 2004) and damage caused by environmental hazards (Jolly and Morimoto 2000; Kregel 2002). Thus, the synthesis of Hsps is very sensitive to abnormal environmental stresses, including extreme heat, harmful chemicals and their metabolites, and other complex environmental situations. Therefore, Hsps may constitute valuable tier-1 biomarkers among the broad-response biomarkers that are being used for preliminary screening of complex environments in *in vitro* cells (Bierkens 2000).

Heat shock protein 70 (Hsp70), one of the main Hsp family members, is highly conserved throughout evolution and plays a key role in protecting cells from environmental insults. In addition, high levels of intracellular Hsp70 in lymphocytes may be a danger marker, as observed in late-stage patients with cerebral infarction (Jin et al. 2004b). Hsp70 has also been reported to distribute in the extracellular space as well, suggesting that it may have different functions in different cellular compartments (Young et al. 2004). For example, the induced Hsp70 in plasma acts as a danger signal to the immune system or disease conditions (Campisi et al. 2003; Jin et al. 2004a, 2004b). It has also been suggested that secreted Hsp70 might play an important role in bacterial infection (Davies et al. 2006). The significance of intra- and extracellular Hsps as responsive biomarkers, either for protection or for danger signaling, in workers exposed to coke oven emissions (COEs) remains unknown. Cell injury induced by COEs includes genotoxicity, oxidative stress, and other types of damage to cells. The generation of oxidative stress, indicated by malondialdehyde (MDA) formation, occurs before and concomitantly with lactate dehydrogenase (LDH) leakage (Thomas and Reed 1988). Subsequently, metabolic oxidative stress can induce the expression of Hsp70 (Lee and Corry 1998).

In a previous study (Xiao et al. 2002), we found that levels of Hsp70 in lymphocytes were negatively correlated with the degree of genotoxic damage in a cohort of 43 workers performing their job at the top of coke ovens, where they were exposed to the highest level of PAHs among coke oven workers. In the present study, we investigated whether the levels of lymphocyte and plasma Hsp70 in 101 workers exposed to different concentrations of complex COEs might serve as responsive biomarkers and whether their levels were correlated with internal exposure levels [urinary 1-hydroxypyrene (1-OHP)], genotoxic damage in lymphocytes (by both comet assay and micronuclei test), and plasma MDA concentration and LDH activity.

## Subjects and Methods

### Study subjects

On the basis of previous environmental monitoring data, a total of 101 healthy male workers—all exposed to COEs in a state-run steel company located in northwest China for at least 5 years—were recruited for this study. These workers had performed duties at different locations in the coke oven factories within the company, including at the top, side, and bottom of the coke ovens and in adjunct workplaces. Subjects who had suffered from infectious diseases or underwent surgery unrelated to their jobs in the previous 3 months were excluded. After the workers provided their written informed consents to participate in the study, we used standardized occupational questionnaires to collect information on demographic characteristics, smoking habits, drinking history, occupational exposure status, and medical history. Finally, each participant donated 6.0 mL of venous blood and 20.0 mL of urine samples at the end of the work shift after overnight fasting. The research protocol was approved by both the Ethics and Human Subject Committees of Tongji Medical College (Wuhan) and Shanxi Medical University (Taiyuan).

### Airborne PAH monitoring

Individual airborne samples were collected from different sites where the participants worked, with an average flow rate of 2.0 L/min for 2–6 hr (240–720 L/sample). Quantitative chemical analyses of 17 PAHs, including 8 that are carcinogenic (Ruchirawa et al. 2002), were performed by high-performance liquid chromatography (HPLC) with fluorescence detectors according to Method 5506 of the U.S. National Institute for Occupational Safety and Health (NIOSH 1998).

### Determination of urinary 1-OHP

We determined urinary 1-OHP by HPLC as described previously (Li et al. 2003) with some modifications (Liu et al. 2006). Briefly, 2.0 mL urine was used for each sample, and the identification and quantification of 1-OHP were based on retention time and peak area measured using a linear regression curve obtained from internal standard solutions. The detection limit of 1-OHP was 0.5 ng/mL; we also used 0.35 ng/mL as the default below 0.5 ng/mL. The valid urine 1-OHP concentrations were expressed as micromoles per mole creatinine.

### Measurement of genotoxic damage in peripheral blood lymphocytes

We measured genotoxic damage to peripheral blood lymphocytes by both the comet assay and the micronuclei test. Lymphocytes from 1.0 mL peripheral venous blood were isolated and suspended in D-Hanks buffer as soon as the blood was drawn. The comet assay was carried out under alkaline conditions using the method previously described by Singh et al. (1988) with some modifications (McKelvey-Martin et al. 1993). More than 50 randomly selected lymphocytes were analyzed for each sample. The nuclei with DNA damage were recorded using a fluorescence microscope, and the images were analyzed by IMI comet analysis software (Zhu et al. 2001). The level of DNA damage was expressed using the means and SDs of Olive tail moment (TM) values. The micronuclei rate in lymphocytes was determined as described by Fenech and Morley (1985). A total of 2,000 cells per sample was counted by a well-trained research assistant. The data were reported as the percentage of micronuclei cells per 1,000 cells.

### Determination of MDA concentration and LDH activity

MDA concentration and LDH activity were measured in plasma using the MDA and LDH activity assay kits (Jiancheng Bio Company, Nanjing, China). The MDA concentration in plasma reflected the degree of oxidative stress and damage to cells, and the plasma LDH activity was used to measure damage to cells (LDH leaked from damaged cells). The results were reported by concentration or activity and calculated as nanomoles per milliliter and units per liter, respectively.

### Detection of Hsp70 in lymphocytes and plasma

Lymphocytes were isolated from about 5.0 mL venous blood. We detected Hsp70 in lymphocytes using our previously described method (Xiao et al. 2002) with minor modifications: we used electrochemiluminescence (ECL) Western blot detection to reveal the presence of Hsp70 instead of 3,3′-diamino-benzidine (DAB). Glyceraldehyde-3-phosphate dehydrogenase (GAPDH) served as the internal control, and levels of lymphocyte Hsp70 were reported as the relative amounts. Plasma was also collected, and Hsp70 levels were measured using an ELISA (enzyme-linked immunosorbent assay) kit (Stressgen Bioreagents Company, Victoria, BC, Canada) and calculated as nanograms per milliliter.

### Statistical analyses

The normality of the data was tested using the One-Sample K-S test, and values of the Olive TM values and plasma Hsp70 were log-transformed to normalize the distribution. We performed one-way analysis of variance (ANOVA) for differences among different exposure groups by age, length of work, cigarette smoking, and 1-OHP. The differences in categorized variables (e.g., current smoking, alcohol drinking status) between different exposure groups were evaluated using the chi-square test. We used multivariate analysis of covariance to estimate the differences in the levels of lymphocyte and plasma Hsp70 between exposure groups, with adjustment for age, length of work, smoking (pack-years), and alcohol consumption. Multivariate linear regression was performed for the trend test with adjustment for the same variables. All statistical tests were two-sided with a significance level of *p* < 0.05 and performed using Statistical Package for Social Sciences software (version 12.0) for Windows (SPSS, Chicago, IL, USA).

## Results

### PAH monitoring and general characteristics of workers

**Figure 1:**
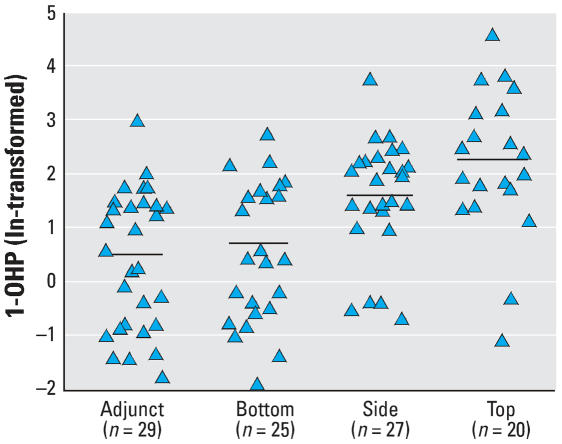
Scatter plot of urinary 1-OHP by different external exposures to PAHs for adjunct work-places and bottom, side, and top of coke oven. Horizontal bars indicate the mean (ln-transformed). Urinary 1-OHP is highest among the workers exposed in the top (2.19 ± 1.36) of the coke oven, and lowest among the workers exposed in the bottom and in adjunct workplaces (0.58 ± 1.31 and 0.41 ± 1.28, respectively).

Table 1 shows the results of airborne monitoring for PAHs at the different work sites of the coke oven. The concentrations of 17 PAHs and the 8 carcinogenic PAHs (mean ± SD) were highest at the top of the coke oven (*n* = 4; 22.83 ± 0.86 and 2.92 ± 0.22, respectively), lower at the bottom (*n* = 4; 6.04 ± 1.85 and 0.42 ± 0.01), and lowest in the adjunct areas (*n* = 8; 5.60 ± 0.87 and 0.41 ± 0.02, respectively). Because all workers had different levels of urinary 1-OHP and there were significant positive correlations between urinary 1-OHP and the exposure to total or carcinogenic PAHs (Figure 1), we defined three exposure groups based on the levels of individual urinary 1-OHP, which included the low-exposure group or the control, intermediate-exposure group, and high-exposure group. The general characteristics of these different exposure groups are presented in Table 2. The mean 1-OHP levels were 0.60 μmol/mol creatinine for the low-exposure group and 13.33 μmol/mol creatinine for the high-exposure group; the differences between these groups were statistically significant in the multivariate linear regression analysis (*p* < 0.001). The distribution of age, length of work, cigarette smoking, and alcohol use in the three exposure groups was similar (*p* > 0.05), and smoking had no significant influence on the levels of urinary 1-OHP in any of the three subgroups (*p* > 0.05; data not shown).

### Dose-dependent increase in cellular damage caused by COEs

We measured levels of damage to lymphocyte DNA as evaluated by comet and micronuclei assays, plasma MDA concentration, and plasma LDH activity (Table 3). Levels of cell injury tended to increase from the low- to high-exposure groups as defined by levels of urinary 1-OHP. Levels of genotoxic damage were lower in the low-exposure group, and the highest levels of cell damage were observed in the high-exposure group, as measured by the comet and micronuclei assays. Both Olive TM (log-transformed values) and micronuclei frequencies were positively correlated with levels of urinary 1-OHP (*p*_trend_ = 0.030 and *p*_trend_ = 0.009, respectively). Moreover, both plasma MDA concentrations and LDH activities were also positively correlated with levels of urinary 1-OHP (*p*_trend_ = 0.010 and *p*_trend_ = 0.015, respectively).

### Levels of Hsp70 in lymphocytes and plasma

**Figure 2:**
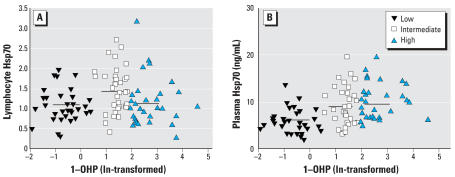
Scatter plots of lymphocyte and plasma Hsp70 to urinary 1-OHP by different internal exposure. (*A*) Lymphocyte Hsp70 (relative amount of GADPH measured by integral optical density) to urinary 1-OHP: the mean levels of lymphocyte Hsp70 were 1.08 ± 0.39 for the low-exposure group, 1.39 ± 0.88 for the intermediate-exposure group, and 1.10 ± 0.55 for the high-exposure group. (*B*) Plasma Hsp70 (geometric mean, ng/mL) to urinary 1-OHP. Plasma Hsp70 levels increased as the exposure increased (*p*_trend_ = 0.044), with the highest plasma Hsp70 (9.74 ± 3.87 ng/mL) in the high-exposure group and the lowest plasma Hsp70 (5.97 ± 2.22 ng/mL) in the low-exposure group.

The mean levels of Hsp70 in lymphocytes and plasma in different exposure groups are also shown in Table 3. The low-exposure group had the lowest levels of lymphocyte Hsp70 (1.08 ± 0.39, mean ± SD), and the workers with the intermediate level of exposure to COEs had the highest mean level (1.39 ± 0.88). Hsp70 in lymphocytes in the workers with the highest exposure (1.10 ± 0.55) was reduced to a level similar to the low-exposure group. Thus, overall there was no significantly linear trend between levels of lymphocyte Hsp70 and levels of exposure (*p* > 0.05) (Figure 2A). However, levels of free plasma Hsp70 increased as levels of the exposure increased (*p* = 0.044); workers with high exposure had the highest mean plasma Hsp70 (9.74 ± 3.87 ng/mL); and workers with low exposure to COEs had the lowest mean plasma Hsp70 (5.97 ± 2.22 ng/mL) (Figure 2B).

### Correlations between Hsp70 and cell injury

Finally, we analyzed correlations between levels of lymphocyte and plasma Hsp70 with levels of cell damage, including DNA damage, micronuclei frequencies, and activities of MDA and LDH, and the data are shown in Table 4. When all workers were pooled together, we found no correlation between these measurements (data not shown). When the workers were divided into the three subgroups according to the levels of urinary 1-OHP, there was still no significant correlation between levels of lymphocyte and plasma Hsp70, genotoxic damage, and MDA concentrations in the low-exposure group; however, we found a positive correlation between plasma Hsp70 and the activity of LDH (*r* = 0.52, *p* = 0.015). Moreover, in the intermediate-exposure group, the level of lymphocyte Hsp70 was significantly correlated with the Olive TM (*r* = −0.56, *p* = 0.048), MDA concentration (*r* = −0.59, *p* = 0.036), and LDH activity (*r* = −0.89, *p* = 0.037). Free Hsp70 in the plasma was only positively correlated with LDH activity (*r* = 0.52, *p* = 0.029). In the high-exposure group, the negative correlation was still observed between lymphocyte Hsp70 levels and Olive TM (*r* = −0.67, *p* = 0.006) and LDH activity (*r* = −0.51, *p* = 0.037). However, the correlation between levels of lymphocyte Hsp70 and micronuclei frequencies was borderline (*r* = 0.34, *p* = 0.080). Compared with the positive correlation between plasma Hsp70 levels and MDA concentration or LDH activity, we found negative trends in correlations between plasma Hsp70 and Olive TM values or micronuclei frequencies in the different exposure groups, although none was statistically significant (*p* > 0.05).

## Discussion

Urinary 1-OHP, a metabolite of PAHs, has been shown to be an indicator of both uptake of pyrene from foods and exposure to exogenous PAHs (Jongeneelen 2001; Kanoh et al. 1993; Ovrebo et al. 1995). Moreover, the level of urinary 1-OHP has been shown to be correlated with genotoxic effects in coke oven workers as determined by a number of assays, including micronuclei frequency and DNA damage measured by sister-chromatid exchange and comet assays (Siwinska et al. 2004). Data from the present study also showed that levels of urinary 1-OHP were associated with exposure to total and carcinogenic PAHs among coke oven workers. Although smoking may also influence levels of urinary 1-OHP (Liu et al. 2006; Zhang et al. 2001), our results did not show any significant difference in 1-OHP levels between current smokers and nonsmokers, which may be due to the fact that levels of PAHs in the workplaces were much higher than those from tobacco smoking. Furthermore, we found that levels of urinary 1-OHP were correlated with levels of DNA damage as measured by micronuclei frequency and the comet assay (Olive TM values), as well as other indicators of cellular damage, such as plasma MDA concentration and LDH activity. Therefore, individual levels of urinary 1-OHP are likely to be a suitable internal indicator of total PAH exposure in the workplaces.

Many recent studies have suggested the possible significance of plasma and lymphocyte Hsps measurements in the understanding of the mechanism of pathogenesis, diagnosis, and prognosis of many diseases (Jin et al. 2004a, 2004b; Radons and Multhoff 2005; Xiao et al. 2002). However, few studies have actually investigated the presence and significance of Hsps in both plasma and lymphocytes either in the same person or in patients with the same disease or exposure to the same environmental stresses. Because Hsp70 can be sensitively induced by a large number of chemicals or metabolites, its presence might be considered an alternative biomarker for exposure to a wide range of pollutants as tested in cultured cells (Bierkens 2000). Our study in workers exposed to COEs further suggests the feasibility of using lymphocyte and plasma Hsp70 levels as response biomarkers.

Data from the present study show that exposure to intermediate levels of COEs induced Hsp70 in lymphocytes. However, Hsp70 levels did not increase further in the high-exposure group, suggesting that such exposure at certain levels might inhibit Hsp70 induction. The expression of Hsp70 in cultured cells is inhibited by benzo[*a*]pyrene (BaP) (Bartosiewicz et al. 2001; Gao et al. 2004), although the exact mechanisms of this inhibition are still not fully understood. Gong et al. (2006) suggested that BaP may suppress the transcription of the *HSP70* gene by reducing the amount of heat shock factor-1 and by decreasing its binding to the heat shock element.

Interestingly, we found significant negative correlations between levels of lymphocyte Hsp70 and the levels of various cell damage in the intermediate or high exposure subgroups. These results suggest that cellular Hsp70 may have played a protective role because further induction could protect cells from genotoxic and oxidative stress damage in the complex coke oven emission stress. It is well known that high emission levels induce lung cancer and chronic obstructive pulmonary diseases (Xu et al. 1996), but whether the decrease in cellular Hsp70 by high exposure levels is involved in the generation of these diseases in coke oven workers remains to be determined. Moreover, we also found that the levels of lymphocyte Hsp70 was negatively correlated with levels of DNA damage as measured by the comet assay but not with micronuclei frequencies induced. The comet assay seems to be a more sensitive method to monitor genotoxic effects than the micronuclei assay; in addition, Hsp70 induction may be an early and sensitive response to cellular stress (Galvano et al. 2002).

The origin of extracellular free Hsp70 in plasma is still unclear; it could be released or secreted by lymphocytes or other organs and tissues in response to abnormal stresses. Our results show that exposure to COEs resulted in a dose-dependent increase in levels of plasma Hsp70. Thus, higher plasma Hsp70 levels may be caused by release rather than secretion of Hsp70 because of increased levels of cell injuries in the highly exposed workers. Indeed, we found positive correlations between levels of plasma Hsp70 and the activity of LDH in the low and intermediate-exposure groups, suggesting the destruction of cellular membrane and/or leakage of cell contents. It is possible that elevation of plasma Hsp70 simply delivers a danger signal of cell damage when the damage is overwhelming compared with the protection role of lymphocyte Hsp70 when the damage is minimal. Although the mechanisms of generation and degradation of plasma Hsp70 are not well understood, several studies have shown that the presence of Hsp70 in the serum was often associated with a poor prognosis of disease (Dybdahl et al. 2005; Park et al. 2006; Pockley et al. 2003). In addition, elevated levels of plasma Hsp70 may exert immune activation as danger signals, as reported in cancer immunity (Radons and Multhoff 2005). High levels were also found to be associated with the occurrence, prognosis, and treatment of heat-induced diseases and heat stroke (Jin et al. 2004a, 2004b; Xiao et al. 2003). Therefore, plasma Hsp70 level may be an effective danger biomarker for evaluating the stress status of workers exposed to COEs.

Using the combination of both lymphocyte and plasma Hsp70 measurements, we found that levels of protection or danger-signal markers were the most significant in the intermediate-exposure group. These data suggest that the highest levels of lymphocyte Hsp70 observed in the intermediate-exposure group may represent a peak of induction, which would then decrease at higher exposure because of the increase in damaged cells; at the same time, the increase of damaged cells that release Hsp70 would cause a small increase of the plasma Hsp70 as the exposure continues, leading to difference in plasma Hsp70 between the intermediate and high-exposure groups. It is possible that in the exposed coke oven workers the balance between the synthesis of lymphocyte Hsp70 and subsequent release was abolished, leading to less Hsp70 release or lower levels of plasma Hsp70 while the levels of damage to the cells may still increase. Another alternative explanation is that the small number in the subgroups may lead to some bias in the data.

In conclusion, in the present study we found different expression patterns of lymphocyte and plasma Hsp70 induced by different levels of exposure to PAHs among coke oven workers. Moreover, results suggest that measurement of lymphocyte Hsp70, preferably combined with plasma Hsp70, may help evaluate individual stress responses either in terms of protection or danger biomarkers among coke oven workers exposed to carcinogenic PAHs. However, more work, with a rigorous design in the exposed population, is needed to establish the utility of the combined levels of lymphocyte and plasma Hsp70 as markers for response to PAHs exposure, including further validation in the exposed populations with larger sample sizes, follow-up visits, and associations with other organic functional abnormalities.

## Figures and Tables

**Table 1:** Levels of external exposure to PAHs among coke oven workers performing their jobs at different locations of the ovens (mean ± SD).

		Coke oven		
PAHs (TWA, μg /m^3^)	Adjunct workplaces (*n* = 8)	Bottom (*n* = 4)	Side (*n* = 4)	Top (*n* = 4)
Naphthalene	3.50 ± 2.05	1.77 ± 0.32	3.95 ± 1.91	10.62 ± 1.34
Acenaphthylene	0.48 ± 0.10	0.53 ± 0.04	1.10 ± 0.00	1.55 ± 0.07
Acenaphthene	0.41 ± 0.09	1.15 ± 0.57	0.90 ± 0.01	1.35 ± 0.07
Fluorene	0.15 ± 0.03	0.77 ± 0.49	0.28 ± 0.00	0.78 ± 0.04
Phenanthrene	0.29 ± 0.07	1.06 ± 0.56	0.62 ± 0.13	2.12 ± 0.46
Anthracene	0.03 ± 0.02	0.12 ± 0.07	0.07 ± 0.01	0.26 ± 0.02
Fluoranthene	0.15 ± 0.03	0.11 ± 0.03	0.39 ± 0.16	1.61 ± 0.18
Pyrene	0.12 ± 0.02	0.11 ± 0.02	0.34 ± 0.15	1.22 ± 0.13
Benzo[*a*]anthracene^a^	0.06 ± 0.01	0.06 ± 0.01	0.16 ± 0.06	0.48 ± 0.03
Chrysene^a^	0.03 ± 0.01	0.04 ± 0.01	0.11 ± 0.09	0.78 ± 0.31
Benzo[*e*]pyrene	0.08 ± 0.02	0.09 ± 0.02	0.17 ± 0.00	0.41 ± 0.05
Benzo[*b*]fluoranthene^a^	0.06 ± 0.01	0.06 ± 0.01	0.12 ± 0.00	0.38 ± 0.11
Benzo[*k*]fluoranthene^a^	0.06 ± 0.01	0.06 ± 0.01	0.12 ± 0.00	0.19 ± 0.02
BaP^a^	0.05 ± 0.01	0.05 ± 0.01	0.10 ± 0.00	0.34 ± 0.02
DB[*a,h*]anthracene^a^	0.03 ± 0.01	0.03 ± 0.01	0.05 ± 0.00	0.10 ± 0.03
Benzo[*g,h,i*]perylene^a^	0.07 ± 0.01	0.07 ± 0.01	0.15 ± 0.00	0.39 ± 0.14
Indeno[1,2,3]pyrene^a^	0.06 ± 0.01	0.05 ± 0.01	0.10 ± 0.00	0.24 ± 0.05
Total	5.60 ± 0.87	6.04 ± 1.85	8.70 ± 2.22	22.83 ± 0.86
Carcinogenic	0.41 ± 0.02	0.42 ± 0.01	0.90 ± 0.03	2.92 ± 0.22

TWA, time-weighted average.

a Known carcinogenic PAHs.

**Table 2:** General characteristics of the coke oven workers in each exposure group.

	Exposure group			
Variable	Low (*n* = 33)	Intermediate (*n* = 35)	High (*n* = 33)	*p-*Value
Age (years)	39.53 ± 1.26	39.16 ± 2.46	39.72 ± 1.99	0.527^a^
Years worked	18.49 ± 3.42	16.72 ± 6.31	18.90 ± 4.98	0.202^a^
Current smokers, yes/no (% yes)	24/9 (72.7)	28/7 (80.0)	25/8 (75.8)	0.778^b^
Cigarette smoking (pack-years)	311.59 ± 260.90	367.09 ± 265.09	317.12 ± 216.17	0.610^a^
Alcohol users, yes/no (% yes)	14/19 (42.4)	13/22 (37.1)	17/16 (51.5)	0.484^b^
1-OHP [μmol/mol creatinine [GM (GSD)]	0.60 (0.25)	4.06 (0.27)	13.33 (0.90)	0.000^a^

Abbreviations: GM, geometric mean; GSD, geometric standard deviation. Values shown are mean ± SD except where indicated.

a One-way ANOVA for differences between the different exposure groups.

b Chi-square tests for differences in the distribution frequencies between the different exposure groups.

**Table 3:** Levels of cellular damage and Hsp70 in lymphocyte and plasma in exposure groups.

	Exposure group				
Cell injury or Hsp70	Low	Intermediate	High	*p-*Value^a^	*p*_trend_^b^
Olive TM (mean ± SD)	1.63 ± 0.46	1.74 ± 0.69	2.54 ± 0.75	0.019	0.030
Micronucleated cells (‰)^c^	2.66 ± 2.09	3.07 ± 2.36	3.85 ± 3.05	0.032	0.009
MDA (nmol/mL)	2.70 ± 0.99	2.60 ± 1.31	3.66 ± 1.82	0.045	0.010
LDH (U/L)	185.97 ± 7.87	221.74 ± 56.14	236.45 ± 120.11	0.044	0.015
Lymphocyte Hsp70	1.08 ± 0.39	1.39 ± 0.88	1.10 ± 0.55	0.026	0.245
Plasma Hsp70, ng/mL [GM (GSD)]	5.97 (2.22)	9.08 (3.25)	9.74 (3.87)	0.012	0.044

Abbreviations: GM, geometric mean; GSD, geometric standard deviation. Values shown are mean ± SD except were noted.

a Multivariate analysis of covariance for the differences between different exposure groups with adjustment for age, length of work, pack-years smoked, and alcohol use.

b Multivariate linear regression for the trend of cell damages with the exposure levels with adjustment for age, length of work, pack-years smoked, and alcohol use.

c Percentage of micronuclei cells per 1,000 cells.

**Table 4:** Correlations of lymphocyte and plasma Hsp70 levels to levels of cell damage in the exposure groups.

	Olive TM		Micronuclei rate		MDA		LDH	
Exposure group	*r*	*p-*Value	*r*	*p-*Value	*r*	*p-*Value	*r*	*p-*Value
Low								
Lymphocyte Hsp70	−0.32	0.117	−0.13	0.550	0.15	0.479	−0.13	0.962
Plasma Hsp70	−0.05	0.877	−0.24	0.346	−0.16	0.514	0.52	0.015
Intermediate								
Lymphocyte Hsp70	−0.56	0.048	−0.23	0.762	−0.59	0.036	−0.89	0.037
Plasma Hsp70	−0.18	0.580	−0.27	0.680	0.26	0.320	0.52	0.029
High								
Lymphocyte Hsp70	−0.67	0.006	0.34	0.080	−0.22	0.405	−0.51	0.037
Plasma Hsp70	−0.16	0.588	0.04	0.895	0.18	0.546	0.36	0.271
